# Variability in phenotype and response to treatment in chronic nonbacterial osteomyelitis; the Irish experience of a national cohort

**DOI:** 10.1186/s12969-021-00530-4

**Published:** 2021-03-25

**Authors:** Daire O’Leary, Anthony G. Wilson, Emma-Jane MacDermott, Clodagh Lowry, Orla G. Killeen

**Affiliations:** 1 UCD Centre for Arthritis Research, School of Medicine, University College Dublin, Dublin, Ireland; 2 National Centre for Paediatric Rheumatology, Children’s Health Ireland, Dublin, Ireland

**Keywords:** Chronic nonbacterial osteomyelitis, CNO, Chronic recurrent multifocal osteomyelitis, CRMO, Autoinflammation

## Abstract

**Background:**

Chronic nonbacterial osteomyelitis (CNO) is an autoinflammatory disease affecting bone with considerable phenotypic heterogeneity and variable association with other autoinflammatory conditions. Disease pathogenesis is incompletely understood, and treatment protocols vary between physicians with no clinical treatment guidelines available prior to 2017. Although CNO was previously considered benign, it is now clear that long-term sequelae do occur.

The aim of this study is to provide a detailed phenotypic description of children and adolescents with CNO who attended tertiary paediatric rheumatology services in Ireland between September 2017 and September 2019, their disease course, treatment and outcomes.

**Methods:**

This study involved retrospective review of clinical notes, laboratory, radiology and histology results of Irish children and adolescents with CNO who are currently attending tertiary paediatric rheumatology services. The Bristol diagnostic criteria were applied retrospectively; only patients who met these criteria were included. Criteria for remission and partial response were based on the Childhood Arthritis and Rheumatology Research Alliance (CARRA) criteria for treatment failure.

**Results:**

Forty-four children and adolescents were recruited. Demographics in terms of age of onset, gender and number of sites were similar to those previously reported. Overall, 18/44 (40.9%) had extraosseous manifestations associated with CNO; 12/44 (27.2%) had cutaneous involvement. All patients received a regular nonsteroidal anti-inflammatory drug (NSAID) after diagnosis with 27/44 (61.4%) requiring at least 1 second-line medication. Second-line agents used in this cohort were bisphosphonates, methotrexate and TNF-blockers. No patients received systemic corticosteroids.

**Conclusion:**

This national cohort showed a high prevalence of extraosseous involvement and a low response rate to NSAID treatment. This may reflect a more inflammatory phenotype and highlights the need to define different subtypes of CNO.

## Background

Chronic nonbacterial osteomyelitis is a rare autoinflammatory condition (OMIM 259680) affecting bone with an estimated prevalence of 1 per 10^5^–10^6^ [1]. It is characterised by relapsing episodes of localised bone pain presenting as pain, swelling or loss of function [2]. Although disease can occur at any site, it predominantly affects the metaphyses of long bones, clavicles, vertebrae and pelvis [2, 3]. Diagnosis is based on clinical presentation with characteristic radiological findings, often supported by histological findings which exclude malignancy and infection [4]. Long-term sequelae include leg-length discrepancy due to growth plate involvement and scoliosis secondary to vertebral body involvement [5–7].

CNO is clinically heterogeneous and variably associated with other autoinflammatory conditions with significant variation in incidence between different cohorts [2, 3, 5, 8–10]. It remains unclear whether the different phenotypes of disease respond differently to treatment. First line treatment with non-steroidal anti-inflammatory drugs has a reported response rate of 50–80% [2, 11]. Second-line treatments include bisphosphonates, disease-modifying antirheumatic drugs (DMARDs), corticosteroids and biologic agents [2, 8, 11, 12]. However, there are no randomised controlled trials to date comparing their relative efficacy in children. Consensus treatment guidelines have recently been produced by CARRA [13] prior to which, second-line treatment was based on expert opinion.

The aim of this study is to describe the characteristics and outcome of patients who attended tertiary paediatric rheumatology services in Ireland. This study provides detailed phenotypic information demonstrating the clinical heterogeneity of this disease in an ethnically homogeneous cohort.

## Methods

Ethical approval for this study was obtained from Children’s Health Ireland (CHI) Research and Ethics committees at Crumlin (REC GEN/572/17) and Temple Street (Reference 14.075). All children and adolescents with a diagnosis of CNO made before 18 years of age who attended the national tertiary paediatric service at two sites in Ireland between September 2017 and August 2019 were invited to participate in this retrospective review. In Ireland, all children with CNO are followed by tertiary paediatric rheumatology services at the two Children’s Health Ireland sites. Potential participants were identified from electronic clinic letters by searching for the terms “CRMO” “CNO” and “osteomyelitis” within the full document text. Written consent and assent was obtained from parents/guardians and participants. Diagnosis was made by a consultant paediatric rheumatologist in all cases based on typical clinical and radiological findings with other diagnoses excluded on histology in the majority of cases. For the purpose of study inclusion, the Bristol Diagnostic Criteria [15] were applied retrospectively (Table 1).

**Table 1:** Bristol Diagnostic Criteria

**1**. Typical clinical and radiological findings in more than one bone (or clavicle alone) without significantly raised inflammatory markers	Typical clinical findings: • Bony pain +/− localised swelling • Absence of local or systemic signs of inflammation or infection
**OR**	Typical radiological findings: • Plain x-rays showing a combination of lytic areas, sclerosis and new bone formation • MRI (STIR) showing bone marrow oedema, bone expansion, lytic areas and/or periosteal reaction
**2**. Typical clinical and radiological findings in one bone plus inflammatory changes (plasma cells, osteoclasts, fibrosis or sclerosis) on bone biopsy with no bacterial growth	

Demographic, clinical, laboratory and radiological information were recorded from the clinical chart. Details recorded included gender, ethnicity, age at onset and at diagnosis, number and site of clinical sites at onset, presence of systemic signs at onset, personal or family history of other inflammatory conditions, disease course and response to treatment (clinical and radiological). Inflammatory markers at presentation (erythrocyte sedimentation rate (ESR) and C-reactive protein (CRP)), antinuclear antibody (ANA) titres and HLA-B*27 status were recorded where available. Histological and microbiological findings were recorded whenever bone biopsy was performed. Regional and whole body magnetic resonance imaging (WB-MRI) findings were recorded for all patients.

Remission was defined as normal ESR, absence of inflammatory pain or clinically active lesions, resolution of marrow oedema on WB-MRI and absence of new lesions on WB-MRI; this definition was based on the CARRA criteria for treatment failure. Partial response was defined as an improvement in clinical findings with a static or improving radiological picture but without full resolution of either. No response was defined as an unchanged clinical and radiological picture or a deterioration in either. Continuous variables were expressed as median and range and categorical as number and percentage (%). Chi-square testing of statistical dependence was performed in RStudio (version 1.1.456).

## Results

Fifty-two patients were invited to participate: no response was received from six patients and two further patients were excluded because they had already transitioned to adult rheumatology services. Data were collected from 44 patients attending tertiary paediatric rheumatology services in Ireland at two sites (CHI at Crumlin, CHI at Temple Street). All participants were of white Irish ethnicity. 32/44 (72.8%) were female. The median age at symptom onset was 9.2 years (range 3.1–14.3 years) and at diagnosis was 10.5 years (range 5.9–15.5 years). Median duration of follow up was 2.97 years (range 0.3–8.3 years). Other clinical and laboratory characteristics are summarised in Table 2.

**Table 2:** Clinical and laboratory characteristics of patients with CNO (*n* = 44)

Characteristic	Number (%)	Median (range)
Female	32 (72.8)	
Age at onset		9.2 years (3.1–14.3)
Age at diagnosis		10.5 years (5.9–15.5)
Diagnostic delay		0.7 years (0.1–2)
Duration of follow-up Presenting symptom:		3.03 years (0.64–8.47)
• Pain	44 (100)	
• Fever	6 (13.6)	
• Clinically unifocal	23 (52.3)	
Total sites (clinical and radiological)		4 (1–19)
Unifocal disease	4 (9.1)	
Extraosseous involvement:		
• Arthritis^a^	9 (20.5)	
• Psoriasis	9 (20.5)	
• Pustulosis	4 (9.1)	
• Aphthous ulceration	4 (9.1)	
• Severe acne	2 (4.6)	
• Pyoderma gangrenosum	2 (4.6)	
• Hidradenitis suppurativa	1 (2.3)	
• Inflammatory bowel disease	1 (2.3)	
Elevated inflammatory markers at presentation		
• ESR	26 (59.1)	
• CRP	10 (22.7)	
HLA B27 (n = 38)	6 (15.7)	
ANA positive > 1:160 (*n* = 38)	4 (10.5)	
Bone biopsy	34 (77.3)	
Family history:		
• Psoriasis	19 (43.2)	
• Inflammatory bowel disease	7 (15.9)	
• Inflammatory arthritis	9 (20.5)	

^a^Arthritis distant from CNO lesions

### Extraosseous involvement

Overall, 18 patients (40.9%) had extraosseous manifestations associated with CNO. 5/18 (27.8%) of these patients had extraosseous manifestations at the time of diagnosis; one each had pustulosis, acne or juvenile idiopathic arthritis and two psoriasis. None of the patients with extraosseous manifestations at the time of diagnosis presented systemically unwell with fever. Of the patients with arthritis distant to the site of CNO osseous lesions, one had polyarticular juvenile idiopathic arthritis which predated the onset of CNO, one developed inflammatory bowel disease (IBD) and IBD-related arthritis, one Behcet’s disease and two psoriatic arthritis during follow-up. Axial arthritis was present in two patients. Twelve patients (27.2%) had cutaneous involvement associated with CNO as summarised in Table 2. Of these, one patient had co-existence of psoriasis, pyoderma gangrenosum, hidradenitis suppurativa and recurrent aphthous ulceration, one had psoriasis and severe acne, and one a recurrent pustular rash and plaque psoriasis. None of those with unifocal disease had extraosseous involvement.

### Laboratory data

Twenty-six patients (59.1%) had an elevated ESR at presentation. Of these, ESR was mildly elevated (< 50 mm/hr) in 19/26 (73.1%), moderately elevated (50-100 mm/hr) in 6/26 (23.1%) and highly elevated (> 100 mm/hr) in 1/26 (3.8%). In the six patients who presented systemically unwell with fever, ESR was normal in 1/6, mildly elevated in 2/6, moderately elevated in 2/6 and highly elevated in 1/6. CRP was less frequently elevated than ESR; 9/10 patients with a raised CRP also had a raised ESR and one did not have an ESR result available. CRP was elevated in 4/6 of the patients who were febrile at presentation. Total white cell count (WCC) was normal in all but one patient; that patient had highly elevated ESR and CRP with a mildly elevated white cell and neutrophil count. Six patients (15.7%, *n* = 38) were HLA-B*27 positive compared to 8.4% of the Irish population [16].

Bone biopsy was performed to exclude infection or malignancy in 34 patients (77.3%). The time from symptom onset to biopsy was variable with a median of 5 months (range 2 weeks – 3 years). Bacterial culture was not performed on four samples. 26/30 (86.7%) showed no evidence of bacterial infection. Three samples had positive culture results which were considered probable contaminants at the time; one had a mixed growth of *Streptococcus mitis* and *Actinomyces*, one had a very scanty growth of *Streptococcus dysgalactiae* and one had a coagulase negative staphylococcus (CONS) in the enrichment fluid only which was felt to be a contaminant. Two patients failed to respond to appropriate antimicrobial treatment; the patient with CONS in the enrichment fluid only was not prescribed antibiotics. The three patients with positive culture results had subsequent WB-MRI demonstrating multifocal lesions; WB-MRI was performed between 1 and 6 months after the bone biopsy in these patients. *Rhodococcus corynebacteroides* was detected on 16S PCR testing in one further culture-negative patient. This patient failed to respond to antimicrobial treatment and had persistent unifocal disease on WB-MRI so repeat biopsy was performed which was culture negative. 16S PCR testing was only performed in 2/34 samples. Histological findings were variable both within and between samples. The primary indication for sampling and histological examination was the exclusion of malignancy or infection rather than the diagnosis of CNO and this was reflected in the reports. Findings excluded malignancy but were often non-specific from a diagnostic perspective. Findings supportive of a diagnosis of chronic inflammation were frequent, with inflammatory cell aggregates, marrow oedema or fibrosis new bone formation or bone remodelling occurring variably within and between samples. The most frequent inflammatory cells identified were plasma cells in 24/34 (70.5%) and mononuclear cells in 14/34 (41%).

### Imaging

**Fig. 1:**
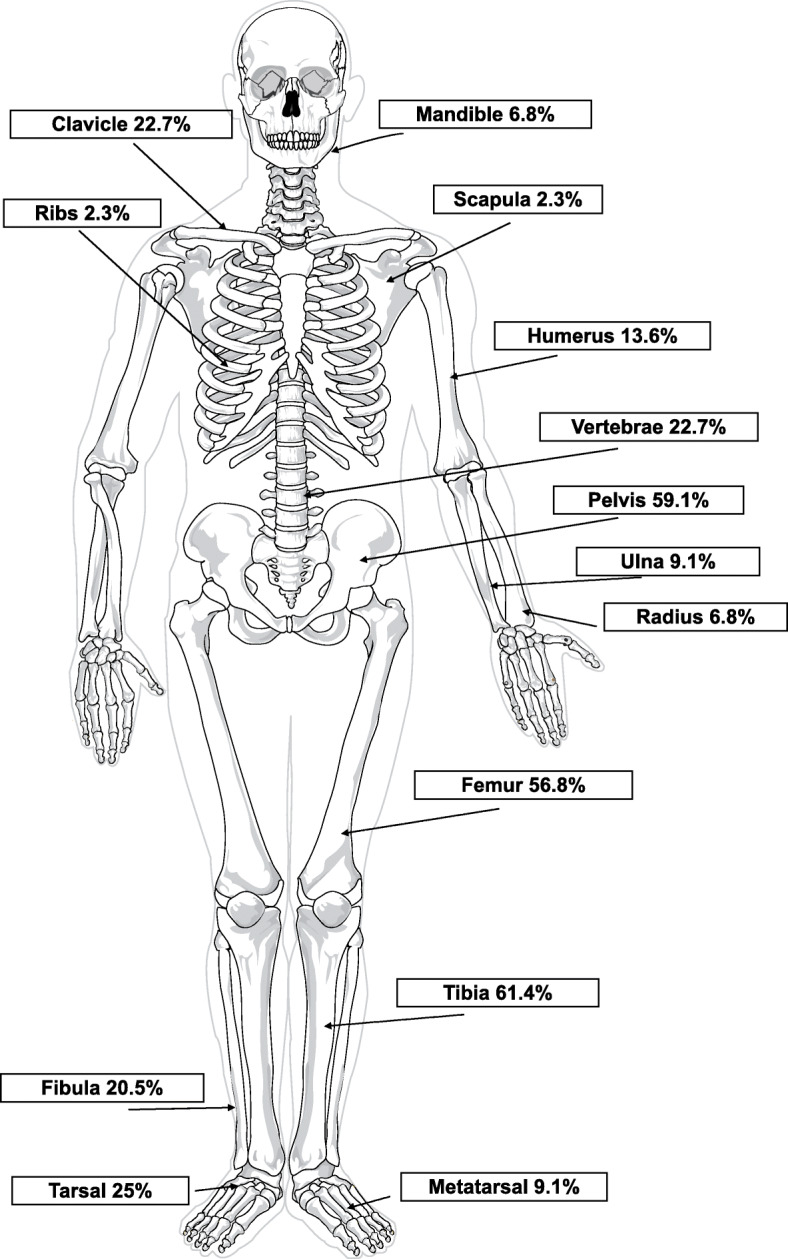
Proportion of patients affected at each site of CNO involvement © Mariana Ruiz Villarreal/Wikimedia Commons/Public Domain

All patients had local plain x-rays +/− local MRI followed by WB-MRI. Follow-up WB-MRI scans were routinely requested every 12 months to assess subclinical disease activity and response to treatment; the frequency of scanning was greater for some patients at certain points in their disease course according to clinical need. The tibia was the most commonly affected bone accounting for 69/251 lesions. In the 4 patients with unifocal disease, 2 patients had clavicular lesions, 1 mandibular and 1 ulnar. Sites involved are summarised in Fig. 1.

### Treatment

All patients received a regular prescribed NSAID following diagnosis. All patients were initially prescribed oral naproxen 10 mg/kg twice daily; subsequent changes to an alternative NSAID depended on patient and physician preference and included a broad range of NSAIDs at appropriate weight-based anti-inflammatory doses. Antibiotic treatment was prescribed to 15/44 (34%) patients prior to diagnosis. Overall, 27/44 (61.4%) patients received at least one second-line treatment. The indications for second-line treatment were poor clinical and radiological response to NSAID after >/= 2 months of treatment (15/27) or the presence of spinal lesions (9/27), lesions affecting the growth plate (1/27) or cosmetically significant mandibular lesions (2/27). 13/27 (48.1%) of patients who received second-line treatment had extraosseous manifestations associated with CNO. Extraosseous manifestations were not the primary indication for second-line treatment in any patient but it is not clear whether the choice of second-line treatment was influenced by the presence of such manifestations. No patients were treated with systemic corticosteroids; this was based on physician and patient preference. Treatment response and remission rates are summarised in Table 3.

**Table 3:** Treatment, response and remission rates

Medication	Number of patients who used (%)	No response (%)	Partial response (%)	Remission (%)
NSAID	44 (100)	27/44 (61.4)	11/44 (25)	6/44 (13.6)
Methotrexate^a^	24 (54.5)	11/24 (45.8)	6/24 (25)	7/24 (29.1)
Pamidronate	6 (13.6)	3/6 (50)	1/6^b^ (16.7)	0
Adalimumab	15 (34)	5/15^c^ (33.3)	6/15 (40)	4/15 (16.7)
Infliximab	7 (15.9)	2/7 (28.6)	3/7 (42.8)	2/7 (28.6)
Etanercept	4 (9.1)	2/4 (50)	1/4 (25)	1/4 (25)

a. Monotherapy; does not include those who received methotrexate as an adjunct to TNF blocker

b. 2 patients have received < 3 months treatment

c. 1 discontinued early due to adverse reaction

Methotrexate monotherapy was administered subcutaneously with a starting dose range of 12.5-15 mg/m^2^. In patients who tolerated treatment but showed no response or a partial response, methotrexate dose was increased to 20 mg/m^2^. Methotrexate-related nausea was the most frequent indication for discontinuation. Nineteen patients received more than one second-line treatment agent. The most frequent progression was from methotrexate monotherapy to a TNF blocker +/− methotrexate in 15 patients. 6 patients received multiple TNF blockers sequentially. The progression and outcome for these patients is summarised in Table 4. Of those patients who received pamidronate, one received it as a second-line agent; all others received it following treatment failure with methotrexate +/− a TNF blocker. There was no statistically significant association between the presence of extraosseous manifestations and the need to progress to second-line treatment (*p* = 0.12).

**Table 4:** Treatment response among patients receiving > 1 TNF blockers

Patient	1st TNF blocker	Indication for discontinuation	2nd TNF blocker	Outcome	3rd TNF blocker	Outcome
1	Adalimumab	Worsening disease	Etanercept	Partial response	NA	
9	Infliximab	Psoriasis	Adalimumab	Remission	NA	
10	Adalimumab	Worsening disease	Infliximab	Anaphylactic reaction	Etanercept	No response^a^
16	Adalimumab	Allergic reaction	Infliximab	Remission	NA	
24	Infliximab	Worsening disease	Adalimumab	Remission	NA	
30	Etanercept	Persistent symptoms	Infliximab	Remission	NA	

^a^Patient 10 had worsening disease on methotrexate and adalimumab, then an anaphylactic reaction to infliximab followed by a brief clinical response to etanercept and then further clinical and radiological progression of disease; this patient is now on Pamidronate and has yet to achieve remission

Clinical course and outcome

Multifocal recurrent disease was the most common subtype in this cohort. 39/44 (88.6%) of patients had a relapsing-remitting multifocal course of disease, 3/44 (6.8%) followed a unifocal recurrent course. Only 2 patients had non-recurrent disease; 1 unifocal and 1 multifocal. Complications occurred in 20/44 (45.5%) patients. The most frequently occurring complication was pain amplification syndrome in 10/44 (22.7%). Prolonged school absenteeism (defined as greater than 20 days absence in one school year after diagnosis) affected 9/44 (20.4%). Vertebral complications, either compression fractures or loss of vertebral height, occurred in 8/10 (80%) patients with vertebral lesions; radiological evidence of these complications was present at the time of diagnosis of the vertebral lesions. None of these patients developed clinically significant scoliosis but 2/8 (25%) had radiological evidence of mild scoliosis. Despite frequent involvement of the long bones of the lower limbs, no patients were affected by leg length discrepancy which was monitored clinically. At the time of last follow-up, 17/44 (38.6%) patients were in remission; 6/17 (35.3%) were in remission on treatment and 11/17 (64.7%) remained in remission after discontinuing treatment. 11/17 patients in remission received second-line treatment with a median duration of treatment of 3.65 years (range 1.5–8 years). Of these 11 patients, 5 discontinued second-line treatment after achieving remission and have remained well to date with a median duration of follow-up of 1 year. Of the patients who have yet to achieve remission, 25/27 (92.5%) had shown a partial clinical and/or radiological response to treatment. 9/25 (36%) of these patients had complete resolution of symptoms with normalisation of inflammatory markers where relevant but subclinical radiological activity. 14/25 (56%) reported some improvement in symptoms with persistent radiological activity. 2/25 (8%) had evidence of improvement but not resolution of both clinical and radiological disease activity. Remission rates for those receiving second-line treatment with and without extraosseous manifestations were comparable at 5/13 (38.5%) and 6/14 (42.3%) respectively.

## Discussion

This study describes a national cohort of patients with CNO with a high response rate to participate. Due to the structure of paediatric rheumatology services in Ireland, all patients with CNO attend tertiary paediatric rheumatology services until remission or transition to adult services. Therefore, this cohort is likely to represent the characteristics of the disease in this country. The demographic characteristics of this national cohort are similar to those previously reported. The presence of an elevated ESR or CRP at presentation in 61.4% of patients is comparable to that previously reported [2, 6, 9, 17]. The incidence of unifocal disease was lower than that seen in most cohorts; the previously reported range is 7–43% [3, 5, 14]. However, all patients in this cohort underwent at least one WB-MRI reducing the risk of undetected asymptomatic lesions [18]. Overall, 30/44 (68.1%) had a personal or family history of psoriasis, inflammatory bowel disease or inflammatory arthritis. In patients, the incidence of mucocutaneous involvement was at the higher end of that previously reported. Psoriasis has been reported 4–22% of patients previously with most studies reporting rates of less than 10% [2, 3, 5, 9, 19–21]. Psoriasis affected 20.5% of the Irish cohort compared with an estimated prevalence of 1.7% in the Irish adult and paediatric population [22]. Of these, 45.4% had a positive family history of psoriasis. It is important to note that all patients in the Irish cohort were under 18 years of age at the time of the study. This contrasts with the previous cohort reporting 22% co-occurrence of psoriasis [5], patients were evaluated as adults and is relevant because only 1/3 of psoriasis has onset in childhood [23]. A further 31.8% of patients had a positive family history of psoriasis in a first- or second-degree relative; overall 43.2% of patients had a positive family history of psoriasis compared to 15–30% previously published [2, 3, 19]. The rate of synovitis and arthritis varies significantly across cohorts ranging from 6 to 56% with 20.5% affected in this cohort [2, 8, 9, 14, 24]. However, whether arthritis is contiguous with or distant to a CNO affected site is not explicit in all studies. In this cohort, only arthritis distant to the site of an osseous lesion was included. The high rate of extraosseous manifestations in this cohort and the frequent family history of autoimmune diseases suggests that this cohort may represent a more inflammatory phenotype of the disease.

Treatment response rates to a regular NSAID and to bisphosphonates were low compared to those previously reported [2, 8, 14, 25, 26]. The lack of even a partial response to bisphosphonate treatment may have influenced physicians’ subsequent treatment decisions when considering treatment options for subsequent patients. It is worth noting that all patients treated with pamidronate had associated inflammatory conditions; 4 had psoriasis, 1 synovitis and pustulosis and 1 Behcet’s disease. It raises the question of whether the low response rates to NSAID and bisphosphonates may be related to the high incidence of extraosseous manifestations although this cohort was too small to demonstrate statistical significance. However, this hypothesis is not supported by previous case series showing a marked improvement in bone pain in paediatric SAPHO patients [27]. Response and remission rates following methotrexate monotherapy were lower than those reported in other cohorts, including the Eurofever registry [2, 14]. Response and remission rates for TNF blockers were similar to those in other cohorts although numbers in all cohorts are small [2, 8, 11]. At last follow-up, 95.4% of patients were clinically improved or in remission. A comparison of remission rates with other cohorts is difficult due to the varying definitions applied. Overall, complications occurred in 43% of this cohort regardless of remission status. Psychosocial complications occurred in 31.8% and were more frequent than physical complications which occurred in 18.1%; physical complications occurred exclusively in those with vertebral involvement. The psychosocial complications were pain amplification syndrome and prolonged school absenteeism which co-existed in 5/14 patients. Prolonged school absenteeism in the years following diagnosis excluded absenteeism for scheduled outpatient care. The prevalence of pain amplification was similar to that seen in a cohort of patients followed to adulthood [5]. However, psychosocial complications were more frequently reported than in other cohorts [2, 3]. Given the variable manner in which outcomes are reported, this may either reflect the more inflammatory phenotype seen in this cohort or under-reporting in some other studies. The variable terminology used to record pain amplification syndrome by clinicians in different specialties may further exacerbate under-reporting of this complication. WB-MRI played an important role in distinguishing amplified pain from CNO-related pain and reduced the risk of over-treatment in these patients.

The retrospective nature of this study is a limitation. This was mitigated in part by only including patients who continued to attend one of the tertiary paediatric centres; thus, some missing data could be clarified during ongoing follow-up. Although the patients’ response rate to the invitation to participate was high - 88% of eligible patients consented to participate – there is a risk of bias and must raise the question of whether those with more severe disease were more likely to participate. In addition, this consisted entirely of patients of white Irish ethnicity. Therefore, these results may not be generalizable to other populations with different or more varied ethnicities. Preferred treatment options varied somewhat between different clinicians. Although objective remission criteria were applied, clinical response was more subjective and dependent upon clinical opinion. In addition, response and remission criteria did not include consistent measurement of response of extraosseous manifestations where relevant.

## Conclusions

The Irish cohort with CNO is associated with high prevalence of extraosseous involvement, a low rate of unifocal disease and a low response rate to treatment with NSAID which may reflect a more inflammatory phenotype of the disease. The significant clinical heterogeneity demonstrated within and between cohorts supports the need to continue to collect consistent data through international collaborations in order to better define different disease subtypes.

## Data Availability

The data and materials from this study can be made available on request.

## Abbreviations

CNO: Chronic nonbacterial osteomyelitis
WB-MRI: Whole body magnetic resonance imaging
CARRA: Childhood Arthritis and Rheumatology Research Alliance
NSAID: Nonsteroidal anti-inflammatory drug
IBD: Inflammatory bowel disease
DMARD: Disease modifying antirheumatic drug
HLA: Human leucocyte antigen
ANA: Antinuclear antibody
CRP: C-reactive protein
ESR: Erythrocyte sedimentation rate
WCC: White cell count
CONS: Coagulase negative staphylococcus
TNF: Tumour necrosis factor
